# Advantages and Limitations of Anticipating Laboratory Test Results from Regression- and Tree-Based Rules Derived from Electronic Health-Record Data

**DOI:** 10.1371/journal.pone.0092199

**Published:** 2014-04-14

**Authors:** Fahim Mohammad, Jesse C. Theisen-Toupal, Ramy Arnaout

**Affiliations:** 1 Department of Pathology, Beth Israel Deaconess Medical Center, Boston, Massachusetts, United States of America; 2 Division of Clinical Informatics, Beth Israel Deaconess Medical Center, Boston, Massachusetts, United States of America; 3 Division of General Medicine and Primary Care, Department of Medicine, Beth Israel Deaconess Medical Center, Boston, Massachusetts, United States of America; 4 Harvard Medical School, Boston, Massachusetts, United States of America; Gentofte University Hospital, Denmark

## Abstract

Laboratory testing is the single highest-volume medical activity, making it useful to ask how well one can anticipate whether a given test result will be high, low, or within the reference interval (“normal”). We analyzed 10 years of electronic health records—a total of 69.4 million blood tests—to see how well standard rule-mining techniques can anticipate test results based on patient age and gender, recent diagnoses, and recent laboratory test results. We evaluated rules according to their positive and negative predictive value (PPV and NPV) and area under the receiver-operator characteristic curve (ROC AUCs). Using a stringent cutoff of PPV and/or NPV≥0.95, standard techniques yield few rules for sendout tests but several for in-house tests, mostly for repeat laboratory tests that are part of the complete blood count and basic metabolic panel. Most rules were clinically and pathophysiologically plausible, and several seemed clinically useful for informing pre-test probability of a given result. But overall, rules were unlikely to be able to function as a general substitute for actually ordering a test. Improving laboratory utilization will likely require different input data and/or alternative methods.

## Introduction

Laboratory testing is the single highest-volume medical activity [1]. Its main role is to help adjust the level of clinical suspicion of a diagnosis to help rule it in or out; it is also used for disease monitoring. In practice, the level of clinical suspicion and the probability of a given test result can be correlated: the higher the suspicion, the more likely it is that the result will confirm the diagnosis. Information that feeds into the clinical suspicion—including the age and gender of the patient, prior diagnoses, and prior laboratory results—thus may also influence the test result.

In principle, this relationship can be used to improve laboratory testing by making it possible to estimate the pre-test probability of getting a given test result before ordering the test, and, in the limit, to reduce test utilization without adversely affecting patient outcomes. Indeed, ordering fewer tests, where warranted, might benefit outcomes by saving the patient the burden of following up false positives (or negatives) [2]–[4].

Conceptually, the relationship between clinical suspicion and pre-test probability is used routinely to help set guidelines regarding when and when not to order a given test. For example, the pre-test probability of Lyme serology being positive given a targetoid rash is high enough that, given the test's sensitivity and specificity, ordering the test is contraindicated [5]. Because of the large number of tests and clinical scenarios that exist, and in light of evidence from across medicine that utilization of laboratory testing can be improved [1], [6], it is of interest to understand whether analyzing large clinical databases using the robust application of standard statistical techniques can turn this relationship into actionable decision-support rules—or whether progress toward better laboratory utilization might instead lie elsewhere.

We sought to test the limits of rule-mining for this purpose. To what extent can laboratory results be anticipated computationally based on data available to the clinician, or a clinical decision support system, at the time of the order? We addressed this question using generalized linear modeling (GLM), a generalized form of linear regression [7], and, for comparison, classification trees (CT) [8], [9].

## Methods

We used four types of input—age, gender, diagnoses (three-digit ICD-9 codes), and results of laboratory tests on blood samples added to the record in the seven days before a given test was ordered—to build simple, robust models for whether the result of a test would be within the reference interval (“normal”) or outside of it in a given direction (“abnormal”), treating high and low results separately.

We based our study on 10 years of records from the Beth Israel Deaconess Medical Center (BIDMC), a 585-bed tertiary care center in Boston, MA. We first anonymized records and reconciled test names (work approved by BIDMC Committee on Clinical Investigation's Institutional Review Board for Research Involving Human Subjects, protocol 2012-P-000229/01). Informed consent was not obtained because patient records/information was anonymized prior to analysis. Each blood test (the test of interest), over 69.4 million in all, was marked as an in-house test (performed at the hospital) or a sendout (performed off-site). For each test, we compiled a list of all instances in which the test was ordered and performed. For each instance, we recorded the patient's age, gender, and any diagnoses or other blood-test test results from the seven days prior to the result of interest. When a test was ordered multiple times within a seven-day period, we considered only the most recent one (i.e., the one closest in time to the sendout order) as input data. For relevance, we considered only those tests that were ordered at least 1,000 times over the entire 10-year period, for an average of at least twice a week. We randomly divided the resulting instances into a training set and a test set (see below for details).

All tests had either two (reference vs. abnormal) or three (low, normal, or high) possible response values. For tests with three values, we performed two separate rule searches: one for high vs. not high—i.e., grouping normal and low—and one for low vs. not low.

### Generalized linear modeling (GLM)

We sought to identify simple, robust subsets of our input data to evaluate as linear predictors (“rules”) for whether a test result would be normal or abnormal. To do this, we used GLM twice: first to find rules based on a particular training set and a second time to find rules based on just those items that were common to rules found from a number of different training sets (to avoid overfitting any one training set). We did this as follows, for each test of interest (the response variable or “response”).

We first excluded those input variables (“features”) that appeared with fewer than 5 percent of the response. We then temporarily set aside the most common features (those of the complete blood count and basic metabolic panel) as well as age and gender, and searched the remaining items for frequent featuresets (using the Apriori algorithm [10], [11]). We then added back to each resulting featureset the common features, age, and gender (which are frequent items by definition, since they appear in all instances) with a support threshold of 0.60 (i.e., itemsets for which all items were present with at least 60 percent of instances of the response variable). This set-aside/add-back approach sped the search for featuresets without loss of comprehensiveness.

We used each featureset to create a model for the test of interest using R's *glm* function (with the family argument set to “Binomial”). We used backward feature elimination to remove non-significant features one at a time from the featureset (using a significance threshold *p*-value of 1×10^−5^; see below) until the only features that remained were all significantly correlated with the response. We also removed features that are used to calculate the result for the test of interest—e.g., CD4 and CD8 count for T-cell count, which is the sum of CD4 and CD8—for all but proof-of-principle runs.

The significance threshold was corrected for multiple comparisons by dividing the traditional threshold of *p* = 0.05 by the product of the total number of tests considered and the average number of rules generated for each test. The combined total number of features (in-house tests plus sendout tests plus diagnoses) was 170+81+434 = 685. The average number of rules after application of GLM for the first time for each test is 6. Thus our threshold *p*-value was 0.05/(6*685) = 1.2×10^−5^, which we rounded to 1×10^−5^.

We constructed a model for the result by running *glm* a second time on a training set (see below) based on this reduced featureset. Of note, there was no guarantee that any feature would be significantly correlated (*p*≤1×10^−5^) or that there would be enough instances (*glm*'s threshold was 200) of the test appearing with all features of even the reduced featureset for *glm* to produce a model. When feature elimination resulted in no significant features or too few instances, no model was constructed. We scored models using PPV, NPV, and ROC AUC.

**Figure 1:**
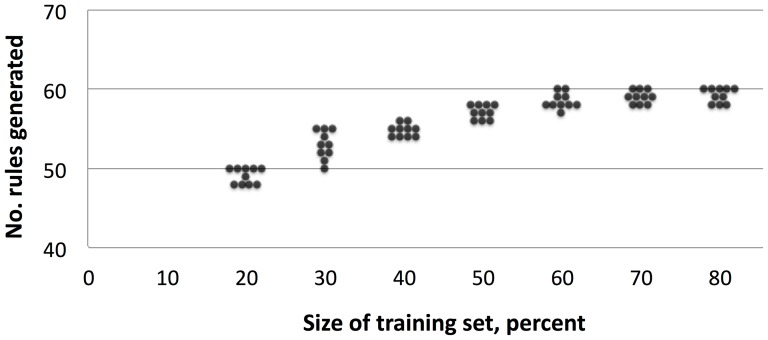
Performance as a function of training set-test set split. A 60-40 split generated a total number of rules comparable to 70-30 and 80-20 splits but with less training data.

We were interested only in models that were robust to the size and choice of training set. Therefore we repeated the above process for a range of training set-test set splits (80-20, 70-30, 60-40, 50-50, 40-60, 30-70, and 20-80 percent). For each split, we ran the above process 10 times and found the number of rules with AUC≥0.75. We decided on using a 60-40 split for downstream analyses as this split generated a total number of rules comparable to 70-30 and 80-20 splits but with less training data (Fig. 1).

Finally, for each test of interest, we selected features that appeared in a strict majority of rules for that test and reran *glm* using only those features. This made rules both simpler and more robust by removing features whose presence was contingent on a particular choice of training or test set.

### Classification and Regression Trees (CART)

For each of the inhouse and sendout tests we used CART, implemented as *RPART* in R (*rpart* v3.1-50; CRAN.R-project.org/package = rpart), to predict the response from all input features, using 60∶40 training∶test-set splits. We fixed some of the metrics (see below) that were used in building the final tree. The CART grows classification tree in two stages. In stage one, a tree is grown by finding a feature which best splits the data into two groups. Splitting is done only if the overall “impurity,” the number of outcomes different from the majority (e.g., a “low” response alongside many “normal” responses), decreases, above some threshold (the “complexity parameter;” 0.01). Then, in top-down fashion, these two subgroups are further divided in a recursive manner until the subgroups reach a minimum size (minsplit = 20 records) or until no further improvement can be made. The resulting tree may overfit the training data. To avoid this, cross-validation (xval = 10; 10-fold cross-validation) was used in the second stage by pruning the tree. We fixed the maximum depth (maxdepth) of the tree, i.e., the maximum number of branchings from stem to leaf, to be 20. The final models were tested on the test data and performance statistics are found. We repeated model-building 10 times for each test and summarized the statistics.

Data-processing was performed in Python (Enthought Canopy Python version 2.7.3. R (version 2.15.3) was used for statistical analysis and reports generation.

## Results

To determine how well sendout and in-house test results can be anticipated based on basic information available in the medical record, we used two independent methods—generalized linear modeling (GLM) and classification and regression trees (CART)—to build simple, robust test-result predictors and then evaluated the performance of these predictors according to the standard clinical metrics of positive predictive value (PPV) and negative predictive value (NPV), as well as sensitivity and specificity via the receiver-operator curve (ROC) area under the curve (AUC).

As proof of principle for GLM, we first tested it on the anion gap, a result calculated by subtracting the serum concentrations of the anions chloride and bicarbonate from those of the cations sodium and potassium, and confirmed that our methods found a rule for elevated anion gap based on these four items.

We next applied GLM to 81 sendout tests ordered regularly at our hospital. GLM generated rules for just 11 of these tests. For the remaining tests, either no recent diagnosis or in-house test result (or age or gender) was sufficiently correlated with the sendout test result, or there were not enough instances in which correlated items appeared with the result, to generate a rule. Only two tests—for high corticotropin (ACTH) and for low ceruloplasmin—had NPV≥0.95. Of these, ceruloplasmin had a PPV≥0.94. The mean AUC for all rules was 0.69, with models for only three tests having an average AUC≥0.75 over 10 repeat runs. Removal of features that did not appear in a majority of rules had essentially no effect on these AUCs (difference in mean AUC≤0.02).

CART generated rules for 60 tests. However, the AUC for most of these rules was low, with only five tests having AUC≥0.75: free T3, alpha-macroglobulin, CA27-29, hyaluronic acid, and alpha fetoprotein (AUC 0.75–0.79).

We next applied GLM to in-house tests. A total of 170 in-house tests were analyzed. A number of rules exhibited a high PPV (the probability of seeing an abnormal value given a prediction of an abnormal value by the rule) or NPV (the probability of seeing a normal value given prediction of a normal value). These were mostly components of the complete blood count (CBC) and metabolic panels. Interestingly, the predictive power of these rules was almost exclusively based on a previous measurement of the test in question: in other words, the best rules were for repeat tests, and the best predictor of a result being normal or abnormal was whether it had been normal or abnormal within the previous seven days. For example, the NPV for a low red blood cell count was 0.95 (with PPV = 0.75), with a rule that depended most on the previous red blood cell count also having been low, and the PPV for high total calcium was 0.98 (NPV = 0.76) and based exclusively on the previous total calcium having been high.

**Figure 2:**
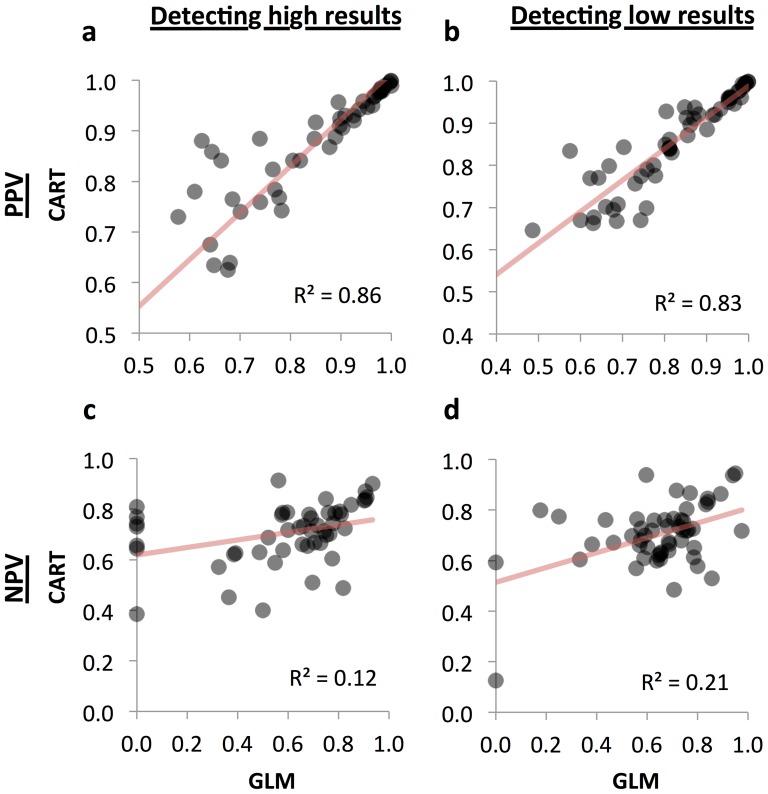
PPV and NPV for the same test, GLM vs. CART. Both linear modeling (GLM) and classification trees (CART) were better at finding rules with high positive predictive value (PPV; panels a and b), with good agreement between the methods, than negative predictive value (PPV; panels c and d).

For comparison, we applied CART to in-house tests, again including in the input data the most recent result for that test if performed within a week of the order. Again, a number of rules exhibited a high PPV (≥0.95), and again these were often tests of the CBC and metabolic panels, with rules based almost exclusively on a previous abnormal value. Examples included low white blood cell count (WBC; PPV = 0.97, NPV = 0.79), platelet count (0.95, 0.88), and serum sodium (0.96, 0.65), and high total calcium (0.99, 0.67), mean corpuscular volume (0.98, 0.84), and iron (0.97, 0.56) all of which were determined almost exclusively from the previous value being low or high (Table 1). Overall, there was good agreement in PPV between GLM and CART for tests for which both methods found rules, but CART outperformed GLM noticeably in NPV (Fig. 2).

**Table 1:** PPV, NPV, and key predictors for selected tests.

GLM				
Test		PPV	NPV	Key predictors
high	anion gap	0.98	0.58	anion gap
high	Bicarbonate	0.95	0.76	bicarbonate, creatinine, heart failure
high	total calcium	0.99	0.73	total calcium
high	MCV	0.98	0.90	MCV
high	potassium	0.97	0.32	potassium
low	alkaline phosphatase	0.99	0.79	alkaline phosphatase
low	MCV	0.99	0.84	MCV
low	potassium	0.96	0.32	potassium
low	BUN	0.98	0.74	gender, BUN
low	WBC	0.96	0.84	WBC

CART				
Test		PPV	NPV	Key predictors
high	anion gap	0.98	0.64	anion gap
high	bicarbonate	0.95	0.79	bicarbonate
high	total calcium	0.99	0.67	total calcium
high	RDW	0.95	0.95	RDW
high	sodium	0.98	0.65	sodium
high	free T4	0.95	0.80	free T4
high	transferrin	0.98	0.39	transferrin
high	anti-cardiolipin IgG	0.95	0.50	thrombin, PTT, WBC
high	free T3	0.96	0.85	free T4
high	prealbumin	0.99	1.00	albumin, PLT
high	SSM antibody	0.96	0.50	C3, age
high	SSA antibody	0.98	0.50	rheumatoid factor, diffuse diseases of connective tissue
low	chloride	0.96	0.68	chloride
low	cortisol	0.96	0.50	cortisol, age, PTT
low	MCV	0.99	0.85	MCV
low	PTH	0.96	0.57	PTH, total calcium, age
low	sodium	0.95	0.72	sodium
low	WBC	0.96	0.83	WBC
low	alpha-1 antitrypsin	0.99	0.00	iron, AST
low	ceruloplasmin	0.96	0.62	transferrin, INR, WBC
low	erythropoietin	0.97	0.64	RBC, PLT, lymphocytes, HCT
low	1,25 vitamin D	0.98	0.14	creatinine, PTH, RBC

The most important key predictors are shown; specifically, those that accounted for at least two-thirds of the predictive power of the rule. Abbreviations: BUN, blood urea nitrogen; HCT, hematocrit; INR, international normalized ratio; MCV, mean corpuscular volume; PTT, partial thromboplastin time; PLT, platelet count; PTH, parathyroid hormone; RBC, red blood cell count; WBC, white blood cell count.

## Discussion

The growing availability of large clinical databases has raised interest in the possibility of using systematic rule-mining for clinical decision support [12]–[15]. One popular and well characterized approach has been logistic regression [16]–[18], a special case of generalized linear modeling (GLM). Researchers have applied these approaches for diverse health-related purposes including prediction of cardiovascular risk [19], mortality in head trauma [18], texture analysis of magnetic resonance images [16], and many other applications [17], [20], [21]. However, we note that GLM does not easily incorporate missing values, as it removes records with missing features; a feature will be “missing” for any record in which that test (the feature) was not performed.

Other methods, such as classification and regression trees (CART) and artificial neural networks [18], have also been applied. Most of these studies were limited in scope to predicting risk of a particular diagnosis. Harper [22] compared four classification techniques (regression, CART, artificial neural networks, and discriminant analysis) on four different datasets and concluded that there was no obvious best choice for their data; while CART performed best, regression was fastest and nearly as good. Similar comparative studies on coronary artery disease [20] and Alzheimer disease [23] indicated that newer algorithms such as ANN and random forests [24] have little advantage over simpler, more traditional approaches. Also, the utility and limitations of these approaches for predicting laboratory results (as opposed to diagnoses) are unclear. However, while CART is both a top performer and overcomes GLM's problem with missing values, it is also more computationally intensive and potentially less sensitive to simple algebraic relationships among features (e.g., among sodium, chloride and bicarbonate and the anion gap). Therefore we chose GLM as a well-understood approach with strong performance and excellent speed, and CART as the best-performing complementary approach for purposes of comparison.

Given the importance of laboratory testing, we asked how much information regression- or classification tree-based rules could provide in assessing the pre-test probability of a test result being abnormal for 251 commonly ordered in-house and sendout tests at our hospital.

Data-mining can sometimes find spurious correlations, artifacts of the particular partitioning of the data into training and test set. To avoid such artifacts, we repeated our regression on multiple independent partitions of the data and kept only items that appeared in a majority of the resulting rules. This safeguard also had the effect of simplifying rules by making each rule dependent on a smaller number of items. As expected, the effect on performance was negligible and dependence on the resulting items was more often clinically and pathophysiologically plausible than rules derived from each run.

When data-mining it is also important to consider the setting. The rules we found do not exist in a vacuum but are “contingent” in the sense that they depend on current clinical practice. Certain tests and panels are ordered in patterns. In a sense, contingency is a form of selection bias: there may well be other diagnoses or test result results that correlate with the result for the test of interest that are not routinely measured according to current best practices. However, as long as the setting in which such rules would be applied is the substantially similar to that in which they were found, selection bias would have little if any effect on finding rules. As long as one is clear that one is looking for relationships in a current practice process, and not among all things that could possibly be measured, any rules that are discovered will by construction be setting-appropriate.

But while our rules appear to be plausible and setting-appropriate, the motivating question behind this study is whether the rules we found could be useful clinically. One way to approach this question is by considering the positive and negative predictive value of each rule (PPV and NPV). These metrics are in contrast to sensitivity and specificity, by which rules are often measured but which do not incorporate disease prevalence in spite of its importance to clinical decision-making. A PPV of 0.95 means that when a rule suggests that the test result will be abnormal, the result actually will be abnormal 95 percent of the time. A NPV of 0.95 means that when a rule suggests that the test result will be normal, the result actually will be normal 95 percent of the time.

We found rules with PPV and/or NPV≥0.95 (by GLM) for only two tests that are sendouts at our hospital—one of which is ceruloplasmin, which we have previously suggested is overordered via chart review [25]. In contrast, for in-house tests we found over a dozen such rules. Interestingly, the main determinant for rules for in-house tests was a normal or abnormal result for the same test within the previous seven days. Although in this study we did not set out explicitly to make a statement about repeat laboratory testing, the appropriateness of which has been investigated elsewhere [4], these results suggest that repeat laboratory testing within one week does not always add information that could not have been anticipated from the previous result. Refining this observation using the same unbiased approach we have followed here is potentially an area for future investigation.

Our results should not be taken as a categorical criticism of repeat testing. First, while the PPV was ≥0.95 in several cases, the NPV was more typically 0.70–0.85. Thus, while prediction that a result will be abnormal may be correct 95 percent of the time, which may be good enough to discourage repeat ordering, prediction that a result will be normal may not be so dependable. Therefore use of a rule depends on the subtle distinction of whether the clinical question is “will the result be abnormal” vs. “will the result be normal.” Second, we note that no rules with such strong performance were found for the majority of our sendout or in-house tests by either of our two complementary approaches. Thus while the rules we found can inform clinical decision-makers, the information they provide rarely replaces the information obtained from actually performing these tests.

It is interesting to note that on average, our simple rules yielded a PPV of 0.84 and an NPV of 0.75. This means that on average, rules will correctly predict an abnormal laboratory result 5 times out of 6 (5/6≈0.84) and correctly predict a normal result 3 times out of 4. While not good enough to replace testing (especially for rules that depend on previous test results), these observations raise the question of how much better prediction can get. Integration of information not considered in the present study, including vital signs, chief complaints, and physical findings, may improve prediction by these methods.
